# A Hidden Complication of Pigtail Catheter Insertion

**DOI:** 10.5811/cpcem.2019.11.44913

**Published:** 2020-01-21

**Authors:** Júlio C. Garcia de Alencar, Millena G. Pinheiro Costa, Rodrigo A. Brandao Neto, Heraldo Possolo de Souza

**Affiliations:** São Paulo University, Department of Emergency Medicine, São Paulo, Brazil

## Abstract

Pigtail catheters have emerged as an effective and less morbid alternative to traditional chest tubes for evacuation of pleural air. Rare complications in the literature have been reported. We report a case of a 92-year-old male who presented with dyspnea and shock, noted to have a pneumothorax requiring tube thoracostomy. Computed tomography demonstrated pigtail within the lung parenchyma. We discuss the implications of this occurrence.

## CASE PRESENTATION

**Image 1:**
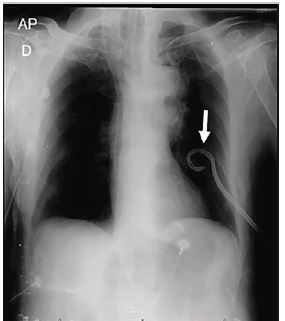
Chest radiograph: well-positioned catheter and good lung expansion (arrow).

A 92-year-old male presented to the emergency department with a 4-day history of flu-like symptoms and shortness of breath that had progressed to respiratory failure. On admission, he was intubated and on vasopressors due to circulatory shock. Endotracheal intubation had been performed at an outside facility; mechanical ventilation was reportedly difficult. At examination, no breath sounds were audible on the left side, and the jugular veins were distended. Point-of-care ultrasound showed no lung sliding on the left. Tube thoracostomy was performed and a pigtail catheter placed, with positive air drainage. Chest radiograph showed a well-positioned catheter and good lung expansion (Image 1).

**Image 2:**
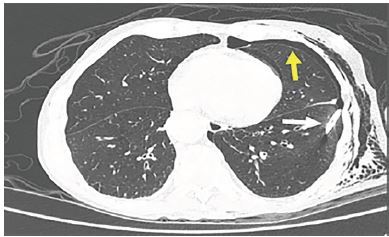
Chest computerized tomography: pigtail within the lung parenchyma (white arrow) and a residual pneumothorax (yellow arrow).

On day two, the patient was extubated and transferred to the Intermediate Care Unit. Three days later he had abrupt onset of dyspnea, extensive subcutaneous emphysema, and drainage of serosanguinous fluid through the chest tube instead of air. Chest computerized tomography showed the pigtail within the lung parenchyma and a residual pneumothorax (Image 2).

The decision was to remove the pigtail and place a traditional chest tube. The patient had an uneventful course, with complete resolution of pneumothorax. The chest tube was removed after six days, and the patient was discharged without further complications.

## DISCUSSION

Pigtail catheters offer reliable treatment of pneumothoraces, and are a safe and less invasive alternative to tube thoracostomy.^1^ Rare complications in the literature such as intraparenchymal insertion, left ventricular penetration, subclavian artery laceration and cerebral air embolism have been reported.^2^ Image guided technique, ideally ultrasound, should be utilized for pig tail insertion to minimize the risk of complications.^3^

**CPC-EM Capsule**

What do we already know about this clinical entity?

Pigtail catheters offer reliable treatment of pneumothoraces, and are a safe and less invasive alternative to tube thoracostomy. Rare complications have been reported.

What is the major impact of the image(s)?

We discuss the implications of this occurrence and recommended management based on our experience.

How might this improve emergency medicine practice?

Image guided technique, ideally ultrasound point-of-care, should be utilized for pigtail insertion to minimize the risk of complications.
